# Truncated NS1 Influenza A Virus Induces a Robust Antigen-Specific Tissue-Resident T-Cell Response and Promotes Inducible Bronchus-Associated Lymphoid Tissue Formation in Mice

**DOI:** 10.3390/vaccines13010058

**Published:** 2025-01-10

**Authors:** Anna-Polina Shurygina, Marina Shuklina, Olga Ozhereleva, Ekaterina Romanovskaya-Romanko, Sofia Kovaleva, Andrej Egorov, Dmitry Lioznov, Marina Stukova

**Affiliations:** Smorodintsev Research Institute of Influenza, The Ministry of Health of the Russian Federation, Saint-Petersburg 197022, Russia; ma.shuklina@yandex.ru (M.S.); ozherelieva.o@gmail.com (O.O.); romromka@yandex.ru (E.R.-R.); so.kovaleva@mail.ru (S.K.); aevirol@gmail.com (A.E.); dmitry.lioznov@influenza.spb.ru (D.L.); marina.stukova@influenza.spb.ru (M.S.)

**Keywords:** influenza, intranasal immunization, truncated NS1 protein, LAIV, universal influenza vaccine, tissue-resident T cells, T follicular helper cells, iBALT

## Abstract

Background: Influenza viruses with truncated NS1 proteins show promise as viral vectors and candidates for mucosal universal influenza vaccines. These mutant NS1 viruses, which lack the N-terminal half of the NS1 protein (124 a.a.), are unable to antagonise the innate immune response. This creates a self-adjuvant effect enhancing heterologous protection by inducing a robust CD8+ T-cell response together with immunoregulatory mechanisms. However, the effects of NS1 modifications on T-follicular helper (Tfh) and B-cell responses remain less understood. Methods: C57bl/6 mice were immunised intranasally with 10 μL of either an influenza virus containing a truncated NS1 protein (PR8/NS124), a cold-adapted influenza virus with a full-length NS1 (caPR8/NSfull), or a wild-type virus (PR8/NSfull). Immune responses were assessed on days 8 and 28 post-immunisation by flow cytometry, ELISA, and HAI assay. Results: In this study, we demonstrate that intranasal immunisation with PR8/NS124 significantly increases tissue-resident CD4+ and CD8+ T cells in the lungs and activates Tfh cells in regional lymph nodes as early as day 8 post-immunisation. These effects are not observed in mice immunised with caPR8/NSfull or PR8/NSfull. Notably, PR8/NS124 immunisation also leads to the development of inducible bronchus-associated lymphoid tissue (iBALT) in the lungs by day 28, characterised by the presence of antigen-specific Tfh cells and GL7+Fas+ germinal centre B cells. Conclusions: Our findings further underscore the potential of NS1-truncated influenza viruses to drive robust mucosal immune responses and enhance vaccine efficacy.

## 1. Introduction

Influenza is an acute respiratory viral infection that poses a significant threat to public health globally [1]. The continuous antigenic evolution of influenza viruses drives annual epidemics and periodic pandemics, resulting in substantial morbidity, mortality, and economic burden [2].

Vaccination remains the most effective strategy for preventing and controlling seasonal and pandemic influenza [3]. However, inactivated influenza vaccines, which are predominantly administered intramuscularly, induce systemic neutralising antibodies that are highly specific to the vaccine strains and provide limited protection against heterotypic influenza infections [4,5]. In contrast, prior influenza infections or nasally delivered live attenuated influenza vaccines (LAIVs) based on cold-adapted (ca) influenza viruses with a full NS1 protein can enhance heterotypic protection by activating local mucosal immune responses [6,7,8,9]. Mediated by tissue-resident T and B lymphocytes and secretory IgA, these responses are critical for blocking infection at its primary entry point and interrupting pathogen transmission [10,11].

Despite the advantages of LAIVs, their application is limited due to insufficient immunogenicity in adults [12]. A promising strategy to enhance the safety and immunogenicity of LAIVs is to inhibit the immunosuppressive function of the non-structural protein 1 (NS1). NS1 suppresses the production of interferons and other pro-inflammatory cytokines, thereby weakening the innate immune response and impairing the development of virus-specific T lymphocytes [13]. Disabling NS1’s functional activity increases vaccine strain immunogenicity and strongly stimulates both innate and adaptive cellular immunity [14,15,16,17]. The superior cross-protective properties of the truncated NS1 influenza A virus (PR8/NS124) compared to sublethal infection with the wild-type strain have been demonstrated in our previous study. Notably, cross-protection against heterologous influenza viruses was observed following intranasal, but not intraperitoneal, immunization with NS1-truncated influenza viruses, which highlights the essential role of the local mucosal immune response in providing heterotypic protection [14]. Moreover, truncation of the NS1 protein further enhanced the cross-protection promoted by cold-adapted strains [18,19]. A direct comparison of the protective efficacy of the truncated NS1 influenza A virus against both wild-type and cold-adapted strains, along with an evaluation of the underlying immune mechanisms, remains an important area of research.

In this study, we evaluated the local adaptive cellular immune response to immunisation with an influenza virus strain PR8/NS124 carrying a truncated NS1 protein. We compared this immune response to those elicited by a cold-adapted virus with a full-length NS1 protein (caPR8/NSfull) and a sublethal dose of a wild-type virus (PR8/NSfull), focusing on their potential to induce Tfh and B-cell reactions.

## 2. Materials and Methods

### 2.1. Viruses

Three strains of the influenza A/Puerto Rico/8/1934 (H1N1) virus strains were used for mouse immunisation experiments: (1) PR8/NSfull, which encodes the full-length NS1 protein; (2) caPR8/NSfull, a cold-adapted strain [20] generously provided by Dr Irina Isakova-Sivak (Department of Virology, Institute of Experimental Medicine, Saint Petersburg, Russia); and (3) PR8/NS124, a strain engineered via reverse genetics to express an NS1 protein truncated to 124 amino acids [21]. All viral stocks were propagated in embryonated chicken eggs (ECE).

### 2.2. Laboratory Animals

Specific-pathogen-free female C57BL/6 mice, aged 6–8 weeks, were purchased from the Laboratory Animal Nursery Pushchino (Shemyakin and Ovchinnikov Institute of Bioorganic Chemistry RAS, Moscow, Russia). All procedures involving animals were performed in compliance with international regulations (Directive 2010/63/EU) and were approved by the Bioethics Committee of the Smorodintsev Research Institute of Influenza.

### 2.3. Immunisation and Challenge Infection

Mice were divided into groups of 20 animals each and immunised intranasally under light ether anaesthesia with either 3.0 Lg EID_50_ of PR8/NSfull or 6.0 Lg EID_50_ of caPR8/NSfull or PR8/NS124 strains. A 10 µL virus suspension in Dulbecco’s phosphate-buffered saline (DPBS; Biolot, St. Petersburg, Russia) was administered to each mouse, while control groups received an equivalent volume of DPBS.

### 2.4. Viral Infectious Activity Analysis

Viral shedding was evaluated on days 2 and 4 post-immunisation. Mice were euthanised, and nasal turbinates and lungs were collected and homogenised using a TissueLyser II bead homogeniser (Qiagen, Hilden, Germany). Viral titers were determined by titrating tissue homogenates in ECE, followed by hemagglutination assays using a 0.5% suspension of chicken red blood cells. The 50% embryo infectious dose (EID_50_) was calculated using the Reed and Muench method [22], with viral titers expressed as Lg EID_50_/mL.

### 2.5. Enzyme-Linked Immunosorbent Assay (ELISA)

To measure influenza-specific antibodies, including IgG and its subtypes (IgG1, IgG2b, and IgG3) as well as IgA, ELISA was performed. Ninety-six-well plates (NuncMaxisorp, Thermo Fisher Scientific, Waltham, MA, USA) were coated with purified A/Puerto Rico/8/1934 virus at a concentration of 2 µg/mL in DPBS. HRP-conjugated antibodies (Abcam, Waltham, MA, USA) were used for detection, and the reaction was visualised with TMB substrate (BioLegend, San Diego, CA, USA), followed by stopping with 1M H_2_SO_4_. Absorbance was measured at 450/620 nm using a Multiskan SkyHigh microplate reader (Thermo Fisher Scientific).

### 2.6. Hemagglutination Inhibition Assay (HAI)

The hemagglutination inhibition assay was conducted according to a standard protocol described elsewhere [23]. The A/Puerto Rico/8/1934 virus, at a standardised antigen concentration of 4 hemagglutination units per 25 µL, was used. The HAI titre was defined as the inverse of the highest serum dilution that completely inhibited hemagglutination.

### 2.7. Lymphocyte Isolation and Stimulation

The assay was conducted as previously described [14]. Briefly, lung and lymph node (LN) lymphocytes were harvested from mice on days 8 and 28 post-immunisation (d.p.i.). and mechanically dissociated. Lung tissue was additionally digested with collagenase/DNase (Sigma, Saint Louis, MO, USA). Cells were filtered through 70 µm strainers, and erythrocytes were lysed using RBC lysis buffer (BioLegend, San Diego, CA, USA). Prepared single-cell suspensions were seeded at a density of 1 × 10^6^ cells per well in 96-well plates (Nunc, Roskilde, Denmark). For intracellular cytokine staining (ICS), cells were stimulated with 5 µg/mL influenza A NP peptide mixture (PepTivator^®^ Influenza A, Mitenyi Biotec, San Diego, CA, USA) supplemented with the NP_366–374_ peptide (Verta Ltd., Saint Petersburg, Russia) and brefeldin A (BioLegend).

### 2.8. Flow Cytometry

To detect CD4+/CD8+ Trm cells that produce cytokines, cells were stained with CD8-PC7, CD4-PC5.5, CD62L-APC-A750, CD44-KO525, CD103-Violet610, CD69-Violet780, IFNγ-FITC, TNFα-PB450, and IL2-PE antibodies (BioLegend, San Diego, CA, USA) using the Fixation and Permeabilization Solution reagent kit (BD Biosciences, San Jose, CA, USA). The panel of fluorochrome-conjugated antibodies including CCR7-Alexa488, CXCR-3-PE/Dazzle, CD4-PerCP-Cy5.5, CD8-PE/Cy7, PD-1-APC/Cy7, CCR6-BV421, CD44-BV510, ICOS-BV605, CD27-BV650, and CXCR-5-BV785 (BioLegend, San Diego, CA, USA) was used to analyse Tfh cell populations. Cytokine-producing Tfh cells were assessed using CXCR-3-PE/Dazzle, CD4-PerCP-Cy5.5, CD8-PE/Cy7, CD154-APC/Cy7, CD44-BV510, ICOS-BV605, CXCR-5-BV785, IFNγ-FITC, IL-2-PE, and TNFα-BV421 (BioLegend, San Diego, CA, USA). GC B-cell markers were analysed using antibodies against CD19-PB, CD38-PE, CD95(Fas)-PE/Cy7, and GL7-AF647 (BioLegend, San Diego, CA, USA). Dead cells were excluded using Zombie viability markers (BioLegend, San Diego, CA, USA), and non-specific antibody binding was blocked using CD16/CD32 specific reagents (BioLegend, San Diego, CA, USA). Data were acquired on a CytoFlex flow cytometer (Beckman Coulter, Bray, CA, USA) and analysed using Kaluza Analysis 2.2 software (Beckman Coulter, Bray, CA, USA). The gating strategies and representative plots are shown in Supplementary Figures S1–S6.

### 2.9. Statistical Analysis

Data analysis was conducted using GraphPad Prism 10.0 (GraphPad Software, Inc., San Diego, CA, USA). Results are presented as mean ± standard deviation (SD) or standard error of the mean (SEM). Statistical comparisons were performed using one-way or two-way ANOVA, followed by Tukey’s multiple comparison test, with *p* < 0.05 considered statistically significant.

## 3. Results

### 3.1. Safety and Reproductive Activity of PR8/NSfull, caPR8/NSfull, and PR8/NS124 Viruses Following Intranasal Immunisation

**Figure 1:**
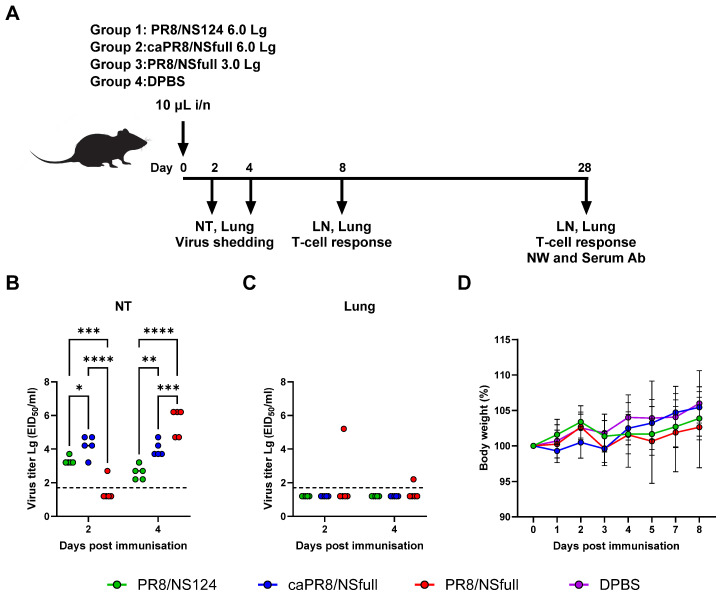
Safety and reproductive activity of PR8/NSfull, caPR8/NSfull, and PR8/NS124 viruses. Study design and sampling plan (**A**). Under light ether anaesthesia, groups of ten 6–8-week-old female C57bl/6 mice were inoculated with 10 μL of either PR8/NS124 or caPR8/NSfull at a dose of 6.0 Lg EID_50_/animal or with PR8/NSfull at a sublethal dose of 3.0 Lg EID_50_/animal. Body weight dynamics were monitored for 8 days after immunisation. Nasal turbinates (NT) and lungs were collected on days 2 and 4 p.im. to assess virus shedding. T-cellular response was evaluated in draining lymph nodes (LN) and lungs on days 8 and 28 p.im. Serum and nasal wash (NW) samples for humoral response assessment were collected on day 28 p.im. Virus shedding in nasal turbinates (**B**) and lungs (**C**). Viral loads in 10% nasal turbinate (NT) and lung suspensions were determined on days 2 and 4 post-immunisation in ECE. Virus titres are expressed as Lg EID_50_/mL. The detection threshold was 1.7 Lg EID_50_/mL (dotted line). If no virus was detected, a value of 1.2 Lg EID_50_/mL was assigned. Mice with a viral load <1.7 Lg EID_50_/mL were considered not infected. Body weight dynamics (**D**) was assessed for 8 days p.im. and expressed as a percentage of the initial body weight (Mean ± SD). Data were considered statistically significant at *p* < 0.05, as determined by two-way ANOVA followed by Tukey’s multiple comparison test (*: *p* < 0.05, **: *p* < 0.01, ***: *p* < 0.001, ****: *p* < 0.0001).

Under light ether anaesthesia, groups of twenty female C57BL/6 mice aged 6–8 weeks were intranasally inoculated with PR8/NS124 or caPR8/NSfull at a dose of 6.0 Lg EID_50_ per animal, or with PR8/NSfull at a sublethal dose of 3.0 Lg EID_50_ per animal (Figure 1A, Supplementary Table S1). The immunisation was performed with 10 μL of inoculum to limit productive infection to the upper respiratory tract. As expected, none of the animals exhibited any clinical symptoms or notable weight loss over the 8-day post-immunisation (p.im.) period. The mean group values for body weight of the immunised animals varied within 5% of the initial body weight and were not significantly different from those of the control group (Figure 1D).

The selected infection dose and inoculum volume were optimised to restrict the replication of all three viruses to the upper respiratory tract. Only the wild-type virus was recovered from lung homogenates in two mice (Figure 1C). The kinetics of viral replication in the nasal cavity varied among the groups. In the PR8/NSfull group, viral titers in nasal turbinates increased from levels near the detection threshold on day 2 to 4.7–6.2 log EID_50_/mL by day 4 post-infection (p.i.). In contrast, the caPR8/NSfull group maintained consistent viral titers in nasal turbinates, ranging from 3.2 to 4.7 log EID_50_/mL at both time points. Notably, the PR8/NS124 group exhibited significantly reduced viral shedding, with viral titers decreasing from a mean of 3.3 log EID_50_/mL on day 2 to 2.6 log EID_50_/mL on day 4 (*p* < 0.0001; Figure 1B).

These findings highlight the reduced reproductive activity and improved attenuation of the PR8/NS124 strain compared to the other two viruses.

### 3.2. Local and Systemic Humoral Responses Following Intranasal Immunization

**Figure 2:**
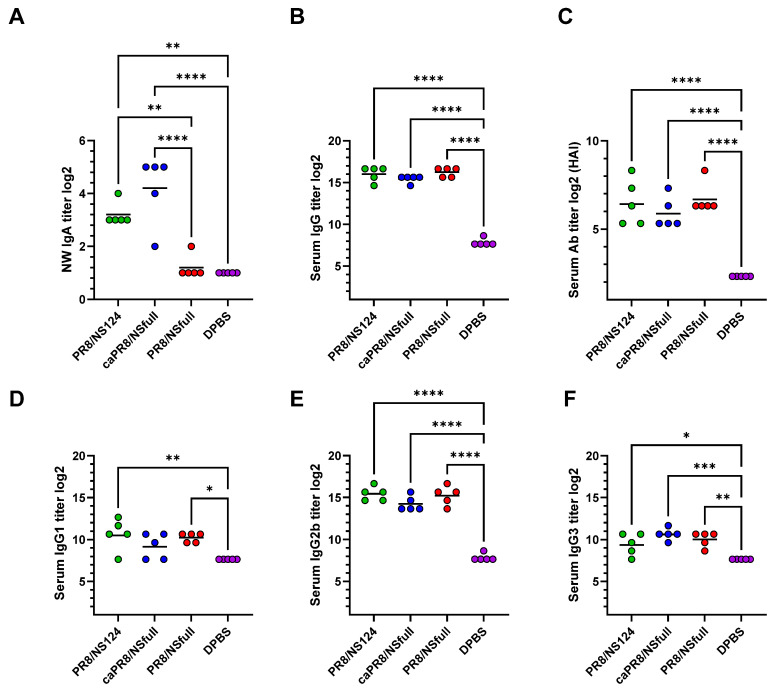
Local and systemic humoral immune responses. Nasal wash IgA titre (**A**), total serum IgG (**B**), anti-hemagglutinating antibodies (**C**), IgG1 (**D**), IgG2b (**E**), IgG3 (**F**). Nasal wash and serum antibodies were measured by ELISA or HAI. Data are presented as individual log_2_ titres with a geometric mean (horizontal line). Data were considered statistically significant at *p* < 0.05, as determined by one-way ANOVA followed by Tukey’s multiple comparison test (*: *p* < 0.05, **: *p* < 0.01, ***: *p* < 0.001, ****: *p* < 0.0001).

Nasal wash IgA and serum antibodies (Ab), including IgG isotypes, were measured 28 days p.im. using ELISA and HAI assays. Despite differences in replicative activity between the strains, all three groups elicited comparably strong systemic humoral responses, predominantly of the IgG2b isotype (Figure 2B–F). However, PR8/NS124 and caPR8/NSfull induced higher local IgA titres compared to PR8/NSfull (Figure 2A).

These results suggest that both PR8/NS124 and caPR8/NSfull have an enhanced ability to stimulate mucosal immunity compared to the wild-type virus.

### 3.3. Tissue-Resident T-Cell Response to Intranasal Immunisation

**Figure 3:**
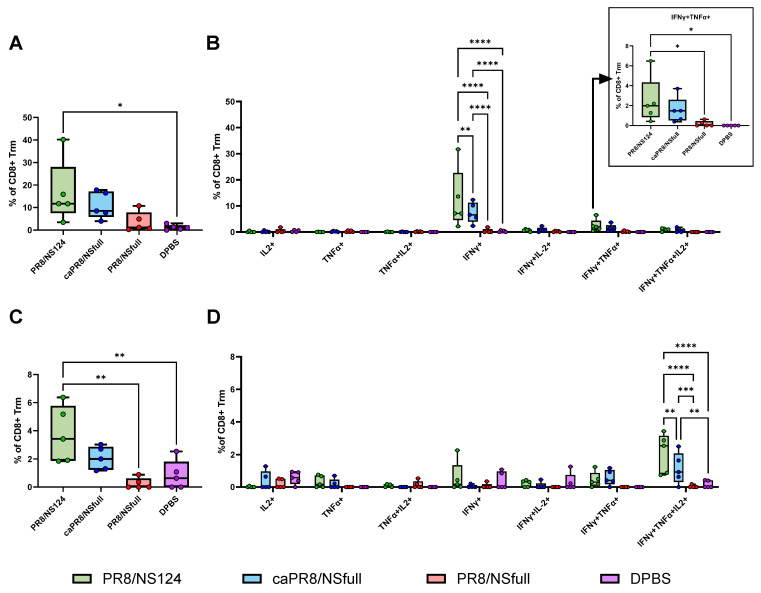
Antigen-specific CD8+ Trm response in the lungs. Trm response in the lungs was assessed at 8 (**A**,**B**) and 28 (**C**,**D**) d.p.im. by intracellular cytokine staining after 6 h of in vitro stimulation with PepTivator^®^ Influenza A (H1N1) NP supplemented with the NP_366–374_ peptide. The total percentage of cytokine-producing CD8+ Trm lymphocytes (**A**,**C**) and the percentage of CD8+ Trm producing any combination of IFNγ, IL2, or TNFα (**B**,**D**) are shown as box and whiskers plots (min and max with individual values and the median indicated). Data were considered statistically significant at *p* < 0.05, as determined by one-or two-way ANOVA followed by Tukey’s multiple comparison test (*: *p* < 0.05, **: *p* < 0.01, ***: *p* < 0.001, ****: *p* < 0.0001).

**Figure 4:**
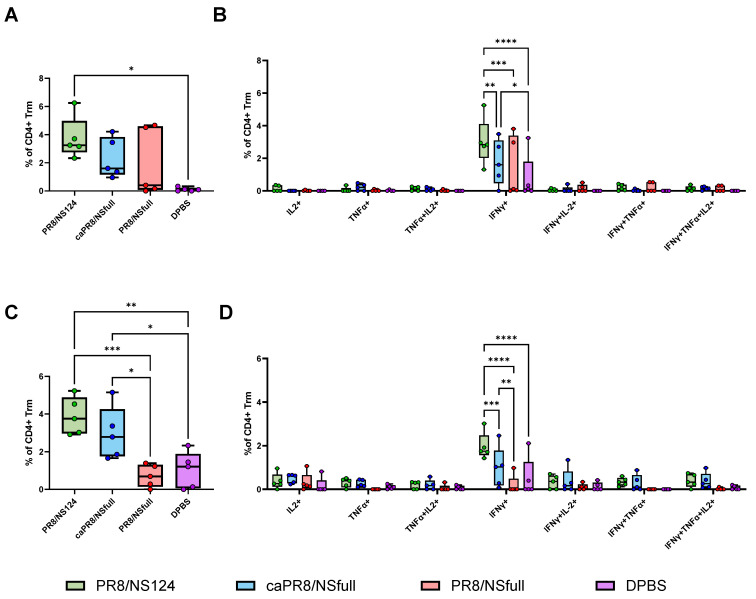
Antigen-specific CD4+ Trm response in the lungs. Trm response in the lungs was assessed at 8 (**A**,**B**) and 28 (**C**,**D**) d.p.im. by intracellular cytokine staining after 6 h of in vitro stimulation with PepTivator^®^ Influenza A (H1N1) NP. The total percentage of cytokine-producing CD4+ Trm lymphocytes (**A**,**C**) and the percentage of CD4+ Trm producing any combination of IFNγ, IL2, or TNFα (**B**,**D**) are shown as box and whiskers plots (min and max with individual values and the median indicated). Data were considered statistically significant at *p* < 0.05, as determined by one-or two-way ANOVA followed by Tukey’s multiple comparison test (*: *p* < 0.05, **: *p* < 0.01, ***: *p* < 0.001, ****: *p* < 0.0001).

To evaluate the tissue-resident memory T-cell (Trm) response, flow cytometry was performed on lung cells using antibodies targeting the surface markers CD4, CD8, CD44, CD62L, CD69, and CD103, as well as cytokines IFNγ, IL2, and TNFα. Subpopulations of Trm cells were identified, and cytokine production was assessed (Figure 3A–D and Figure 4A–D).

The PR8/NS124 group exhibited the most robust Trm response, with the significant formation of antigen-specific CD4+ and CD8+ Trm in the lungs. CD4+ Trm cells were predominantly monofunctional (IFNγ+) at both early (day 8) and late (day 28) time points. In contrast, CD8+ Trm cells showed a dynamic shift: on day 8, they primarily produced IFNγ or IFNγ/TNFα, whereas by day 28, triple-positive polyfunctional IFNγ/TNFα/IL2 cells formed the predominant population.

The PR8/NSfull group induced the weakest Trm response, while caPR8/NSfull elicited a moderate response, surpassing PR8/NSfull but remaining inferior to PR8/NS124.

### 3.4. Tfh Response Activation and iBALT Formation

**Figure 5:**
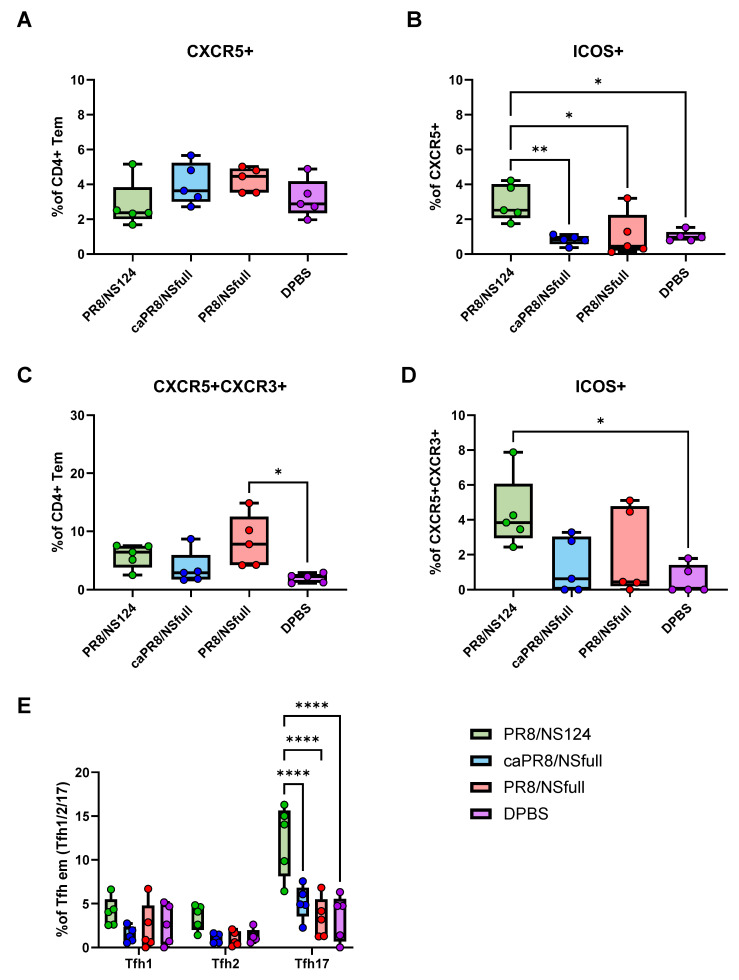
Tfh responses in lymph nodes. Tfh (CXCR5+ or CXCR5+/ CXCR3+) subpopulations were evaluated in lymph nodes at 8 d.p.im. The percentage of Tfh cells in the parent population (CD4+ Tem) (**A**,**C**), ICOS+ Tfh (**B**,**D**), and Tfh1/Tfh2/Tfh17 subsets among lymph node Tfh em (**E**) are shown as box and whiskers plots (min and max with individual values and the median indicated). Data were considered statistically significant at *p* < 0.05, as determined by one- or two-way ANOVA followed by Tukey’s multiple comparison test (*: *p* < 0.05, **: *p* < 0.01, ****: *p* < 0.0001).

To further characterise the CD4+ T-cell response, Tfh cells in draining lymph nodes and lungs were analysed using flow cytometry. Early after immunisation (day 8), activated Tfh cells expressing ICOS were significantly elevated in the PR8/NS124 group compared to other groups (Figure 5B,D). This group also displayed a predominance of Tfh17 cells in lymph nodes at this time point (Figure 5E).

**Figure 6:**
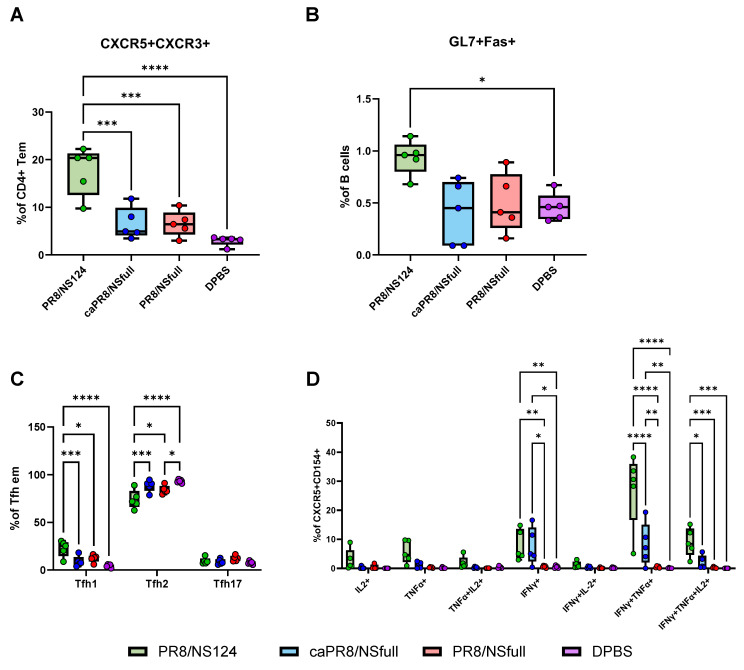
Tfh and germinal centre B-cell responses in the lungs. Tfh subpopulations were evaluated in the lungs at 28 d.p.im. The percentage of Tfh (CXCR5+ CXCR3+) cells in the parent population (CD4+ Tem) (**A**), the percentage of CL7+Fas+ GC B cells (**B**), Tfh1/Tfh2/Tfh17 subsets among lung Tfh em (**C**), and percentages of antigen-specific cytokine-producing Tfh cells are shown as box and whiskers plots (min and max with individual values and the median indicated) (**D**). Data were considered statistically significant at *p* < 0.05, as determined by one-or two-way ANOVA followed by Tukey’s multiple comparison test (*: *p* < 0.05, **: *p* < 0.01, ***: *p* < 0.001, ****: *p* < 0.0001).

By day 28 p.im., PR8/NS124 immunisation led to an increased population of antigen-specific Tfh CXCR3+ cells in the lungs (Figure 6A). This group also had the highest proportion of Tfh1 cells and the lowest proportion of Tfh2 cells among lung Tfh subsets (Figure 6C). Antigen-specific lung Tfh cells co-expressed CD154 with IFNγ, TNFα, and IL2 upon ex vivo stimulation (Figure 6D).

Additionally, PR8/NS124-induced Tfh cells were associated with an elevated percentage of germinal centre B cells (GL7+Fas+), further indicating the presence of inducible bronchus-associated lymphoid tissue (iBALT) in the lungs (Figure 6B).

## 4. Discussion

The immunity elicited by current seasonal inactivated influenza vaccines is strain-specific and offers limited protection against antigenic drift variants of the virus. As a result, influenza epidemics and pandemics remain a significant public health challenge. Cross-protection against a broad spectrum of influenza virus strains requires the stimulation of a localised cellular immune response in the respiratory mucosa. Tissue-resident memory T lymphocytes (Trm)—a key component of mucosal immunity—act as the first and most critical barrier to influenza infection [24,25]. Vaccines capable of inducing Trm formation in the respiratory tract are expected to provide broader protection compared to the currently available inactivated influenza vaccines. However, even though LAIVs can protect against drift influenza viruses of the same subtype, they are insufficient to protect against heterosubtypic variants [26,27].

Deletion of the NS1 protein that antagonises innate and adaptive responses is a strategy to develop an optimal mucosal influenza vaccine. Mucosal influenza vaccines with modified NS1 proteins show great potential as candidates for universal influenza vaccines and as influenza-vectored vaccines targeting respiratory diseases, especially for pathogens with high genetic variability and heterogeneity of circulating virus populations [28,29,30]. To date, several influenza-vectored vaccine candidates against SARS-CoV-2, respiratory-syncytial virus (RSV), and tuberculosis have passed phase I, II and III clinical trials, showing favourable safety profiles and immunogenicity [31,32], (NCT05696067, NCT05970744, NCT05945498).

While the mechanisms of immune responses induced by influenza viruses with modified NS1 proteins have been extensively studied [14,15,16,17,33,34,35,36], their impact on Trm cells and the development of inducible bronchus-associated lymphoid tissue (iBALT) has not been thoroughly investigated. In this study, we compared the immune response elicited by a virus with a truncated NS1 protein (PR8/NS124) to those induced by similar strains with full-length NS1 proteins (caPR8/NSfull and PR8/NSfull).

A significant challenge in immunological studies of LAIVs is determining how to effectively compare viruses with different replication abilities. Therefore, one of the primary objectives of our study was to establish appropriate doses for infecting mice to achieve comparable levels of attenuation and replication in the respiratory tract. Our findings show that intranasal immunisation with 3.0 log EID_50_/mouse of PR8/NSfull and 6.0 log EID_50_/mouse of caPR8/NSfull or PR8/NS124 in a 10 µL volume is well-tolerated in all groups, with no clinical signs of disease or notable weight loss. This approach limits viral replication to the upper respiratory tract. Among the tested strains, PR8/NS124 exhibited the lowest replicative activity, indicating its safety. Despite these differences, all strains elicited robust systemic humoral responses, predominantly of the IgG2b isotype, while local sIgA responses were more pronounced in the PR8/NS124 and caPR8/NSfull groups.

Immunisation with PR8/NS124 resulted in a higher percentage of polyfunctional Trm cells (IFNγ/TNFα+ and IFNγ/IL2/TNFα+) in the lungs compared to caPR8/NSfull and PR8/NSfull. The reduced number of polyfunctional Trm cells in the latter groups may be attributed to the inhibitory effects of the NS1 protein on the RIG-I pathway. Previous studies have shown that impaired RIG-I signalling delays dendritic cell activation and reduces polyfunctional T cell formation [37]. Blocking the immunosuppressive function of NS1 in PR8/NS124 likely enhances the generation of polyfunctional Trm cells, which are crucial for immunity against conserved influenza epitopes [38].

Tfh cells play an essential role in orchestrating long-lasting immunity by regulating B cell responses and facilitating the development of memory B cells [39]. While Tfh cells are typically lymphoid-resident, circulating and non-lymphoid Tfh-like cells have also been observed, particularly in response to vaccination [40,41,42]. The development of tertiary lymphoid structures, such as iBALT, in the lungs is associated with improved host protection against respiratory pathogens, including influenza [43,44]. iBALT supports plasma cell survival and the generation of cross-reactive B cells capable of neutralising antigenic variants [45,46].

The role of different Tfh subpopulations in the immune response is being actively investigated. Tfh1 cells are associated with antigen-specific antibody production in influenza infection and vaccination [47,48] and other viral infections, including SARS-CoV-2 and HIV [49,50]. The predominance of Tfh2 and Tfh17 responses has been linked to the development of allergic pathology and autoimmune disease [51,52]. However, Gao et al. showed that Tfh17 cells were superior to Tfh1 and Tfh2 cells in maintaining Tfh memory, which was attributed to their better survival capacity and higher potential to differentiate into GC-Tfh cells. This phenomenon was consistently observed by the authors in vaccination, infection, and natural antigen exposure [53]. In our study, PR8/NS124 demonstrated superior activation of nodal Tfh cells compared to the full-length NS1 viruses, with an early predominance of the Tfh17 phenotype following immunisation. At later time points, PR8/NS124 promoted the development of iBALT in the lungs, characterised by the largest proportion of Tfh1 cells, high levels of influenza-specific Tfh cells, and germinal centre (GC) B cells. These cellular responses were not reflected in the intensity of systemic and local antibody responses measured on day 28 post-immunisation. Further studies are needed to investigate the possible influence of a more pronounced Tfh response and iBALT formation induced by PR8/NS124 on the duration of humoral immunity and its contribution to enhanced cross-protection provided by viruses with truncated or deleted NS1 proteins.

## 5. Conclusions

Our results suggest that the influenza virus with a truncated NS1 protein elicits robust Trm and Tfh responses in the lungs, contributing to the formation of iBALT. These findings underscore the potential of NS1-modified viruses as promising candidates for mucosal influenza-vectored vaccines against respiratory diseases and universal influenza vaccines, capable of inducing broad cross-protection against antigenic variants. Further research is needed to explore their long-term protective effects and whether this vaccine platform could be translated into practical applications.

## Data Availability

The data presented in this study are available on reasonable request from the corresponding author.

## Supplementary Materials

The following supporting information can be downloaded at: https://www.mdpi.com/article/10.3390/vaccines13010058/s1, Table S1. Experimental design. Figure S1. Trm gaiting strategy. Figure S2. Representative plots of cytokine-producing CD4+ Trm cells. Figure S3. Representative plots of cytokine-producing CD8+ Trm cells. Figure S4. Tfh gating strategy. Figure S5. Representative plots of Tfh cells producing cytokines. Figure S6. GC B-cells gaiting strategy.
