# Supplementary material for: Truncated NS1 Influenza A Virus Induces a Robust Antigen-Specific Tissue-Resident T-Cell Response and Promotes Inducible Bronchus-Associated Lymphoid Tissue Formation in Mice
Source: Vaccines (Basel). 2025 Jan 10;13(1):58. doi: 10.3390/vaccines13010058 (PMC11769193; doi:10.3390/vaccines13010058)

vaccines

MDPI

Supplementary

Table S1. Experimental design.

Group №	Animals	n	Preparation	Dose	Method of administration	Parameters				
						Days 0-8	Day 2	Day 4	Day 8	Day 28
						Body weight	Virus shedding from NT, L		Trm, Tfh response (L, LN)	Trm, Tfh, GC B response (L, LN), Ab response (NW, S)
1	Mice C57BL/6, ♀ 6-8 weeks old	20	PR8/NS124	6.0 lg	i/n, 10 μL	10/20	5/20	5/15	5/10	5/5
2		20	caPR8/NSfull	6.0 lg		10/20	5/20	5/15	5/10	5/5
3		20	PR8/NSfull	3.0 lg		10/20	5/20	5/15	5/10	5/5
4		20	DPBS	-		10/20	5/20	5/15	5/10	5/5
										**Total: 80 mice**

NT - nasal turbinates; L - lungs; LN - lymph nodes; NW - nasal wash; S – serum; Trm - tissue-resident memory T cells; Tfh - follicular helper T cells; GC B - germinal centre B cells; Ab - antibody

The animals were acclimated to the housing conditions in a quarantine room for 7 days. During this period, their clinical condition was monitored through visual inspection, and their body weight was recorded. Each animal was identified with a unique number using ear clips. Animals that did not meet the requirements for their clinical condition, weight (18-20 g), or appearance were excluded from the experiment; however, in this study, there were no cases of exclusion from the experiment. Group assignments were made using a randomisation script in Excel. Before immunisation, the animals were kept in group cages for 3 days to adapt. All procedures involving the animals and subsequent data analysis were carried out under blinded conditions.

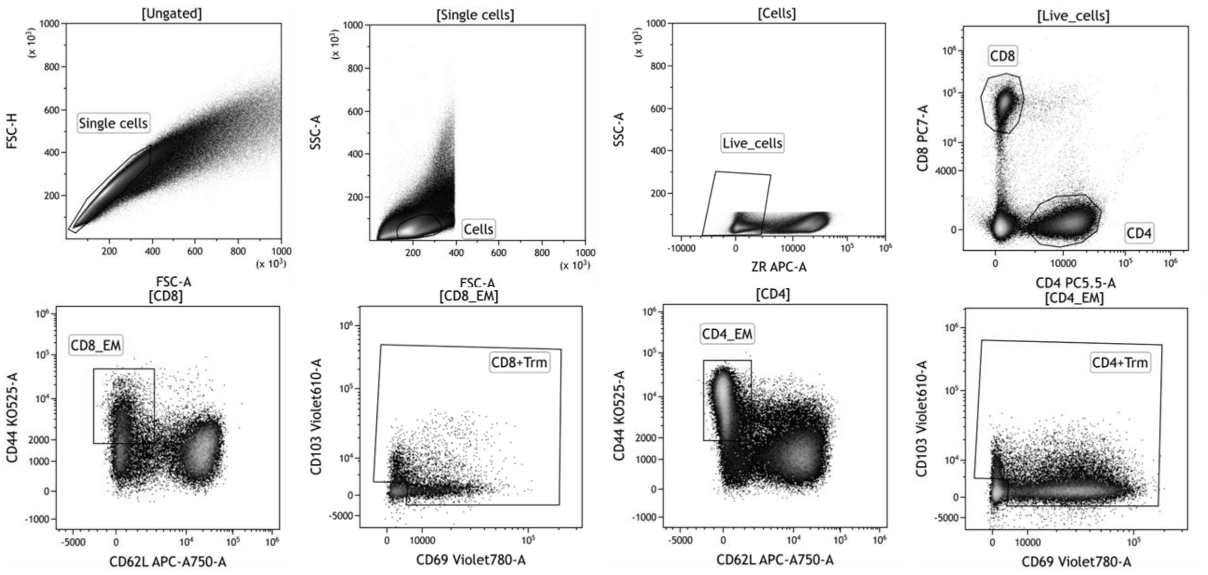

Figure S1. Trm gaiting strategy. Non-viable cells are excluded from the analysis based on forward and side scattered light (FSC/SSC) parameters and the degree of binding of the viability marker Zombie Red. The population of live T lymphocytes is divided into two main subpopulations according to the presence of the surface markers CD4 and CD8: T helper (CD4+) and cytotoxic T cells (CD8+). Subpopulations of naive (CD44-CD62L+), central (Tcm: CD44+CD62L+) and effector (Tem: CD44+CD62L-) memory T cells are distinguished according to the level of expression of the CD44 and CD62L markers. Tissue-resident memory T cells (Trm) are differentiated from effector memory T cells (CD44+CD62L-) based on increased expression of CD69 and/or CD103.

vaccines

MDPI

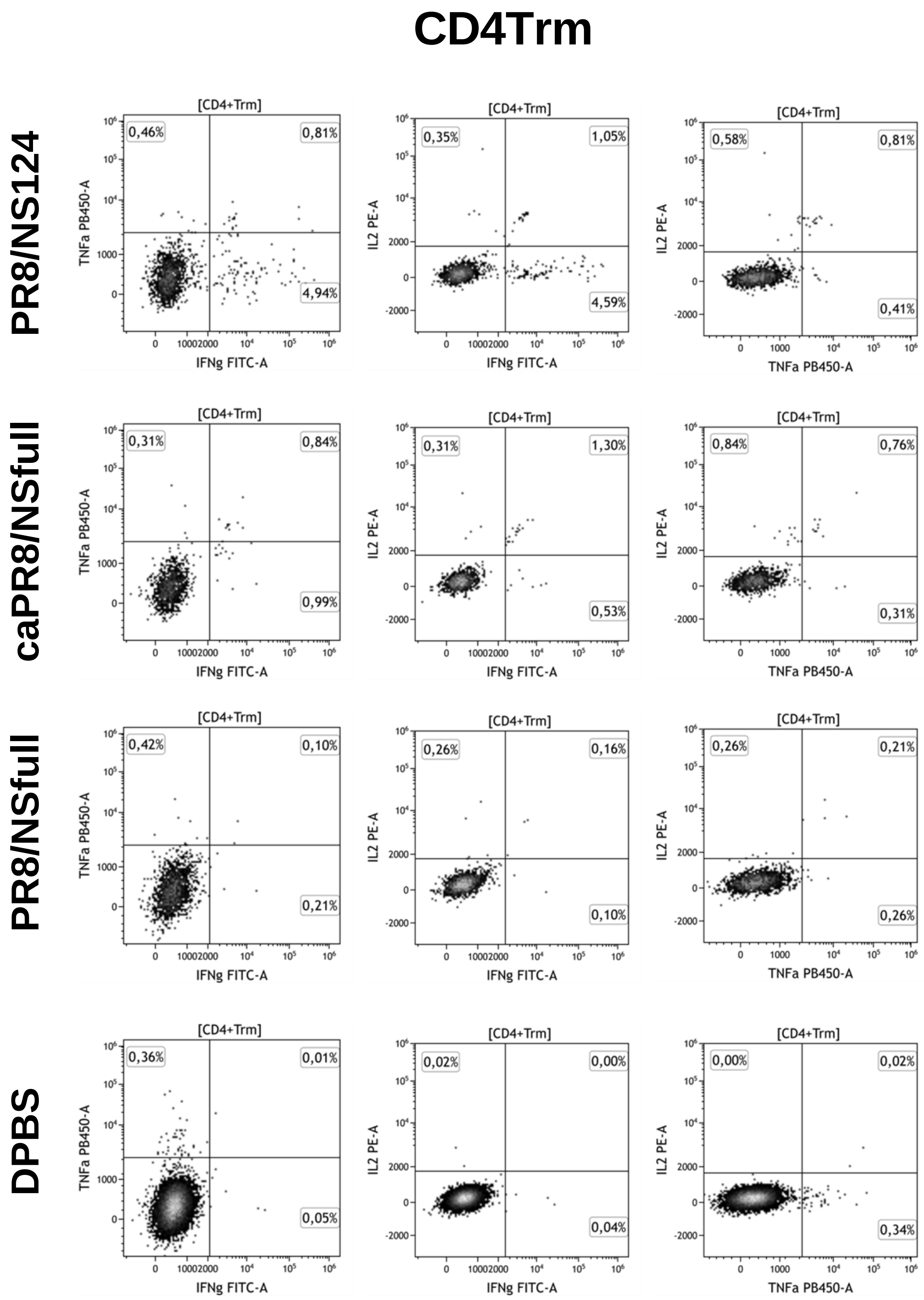

Figure S2. Representative plots of cytokine-producing CD4+ Trm cells

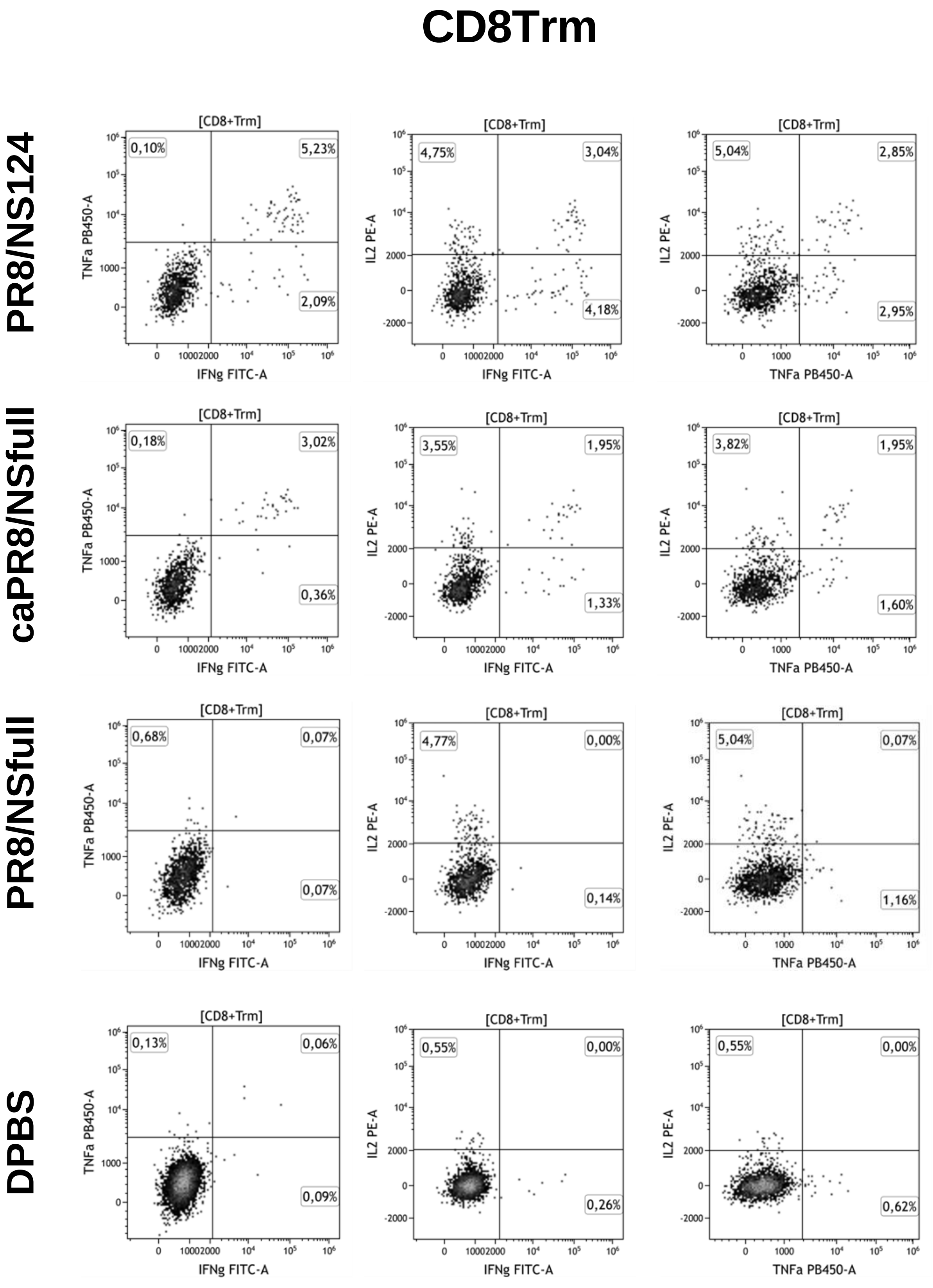

Figure S3. Representative plots of cytokine-producing CD8+ Trm cells

vaccines

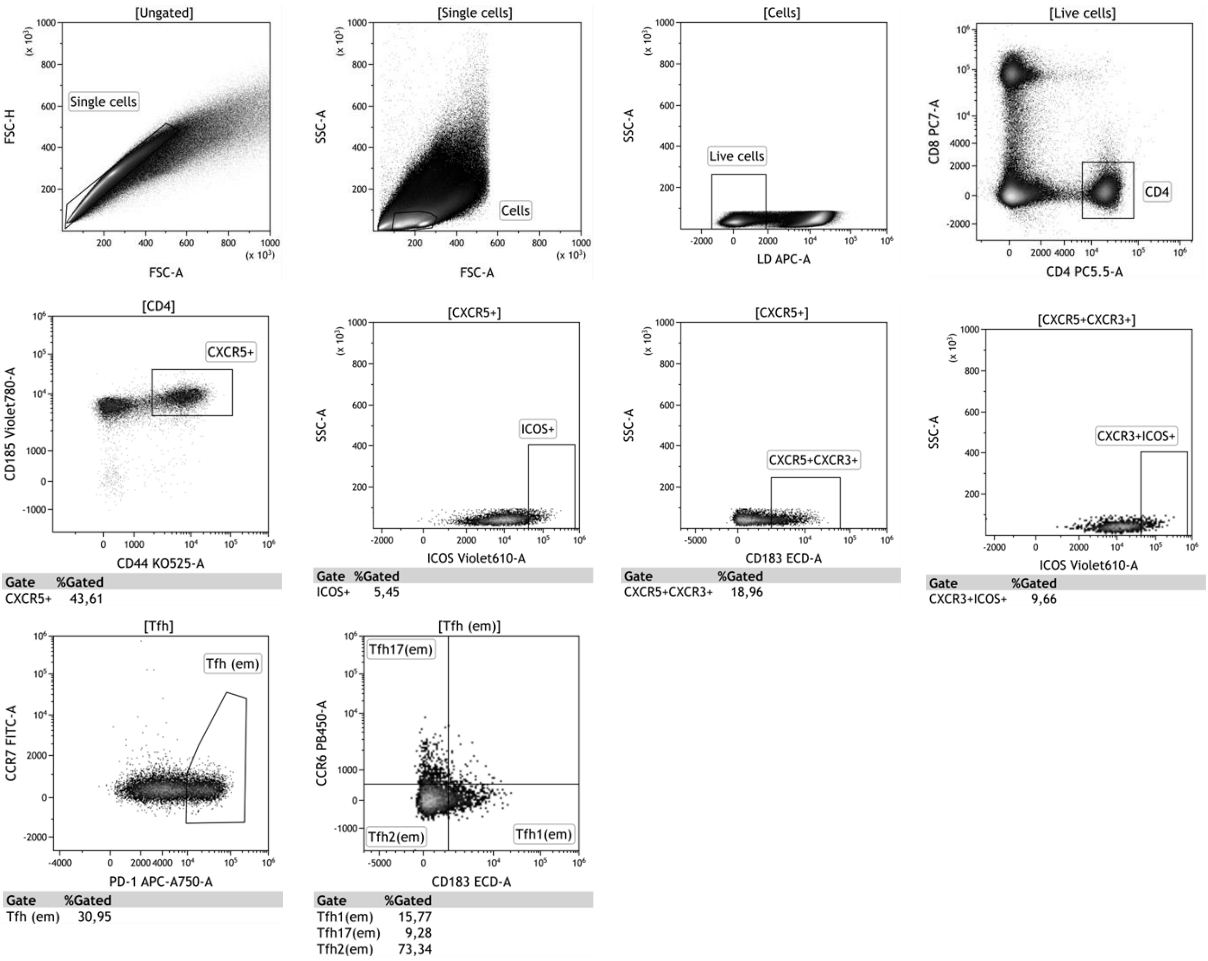

Figure S4. Tfh gating strategy. Non-viable cells are excluded from the analysis based on forward and side scattered light (FSC/SSC) parameters and the degree of binding of the viability marker Zombie Red. The population of live T lymphocytes is divided into two main subpopulations according to the presence of the surface markers CD4 and CD8: T helper (CD4+) and cytotoxic T cells (CD8+). Tfh cells were identified as CD4+CD44+ cells expressing CXCR5 (Tfh) or both CXCR5 and CXCR3 (Tfh CXCR3+). Effector memory (em) Tfh1/Tfh2/Tfh17 subsets within the PD1+ subpopulation of CD4+CD44+CXCR5+ cells were identified based on the differential expression of CXCR3 and CCR6.

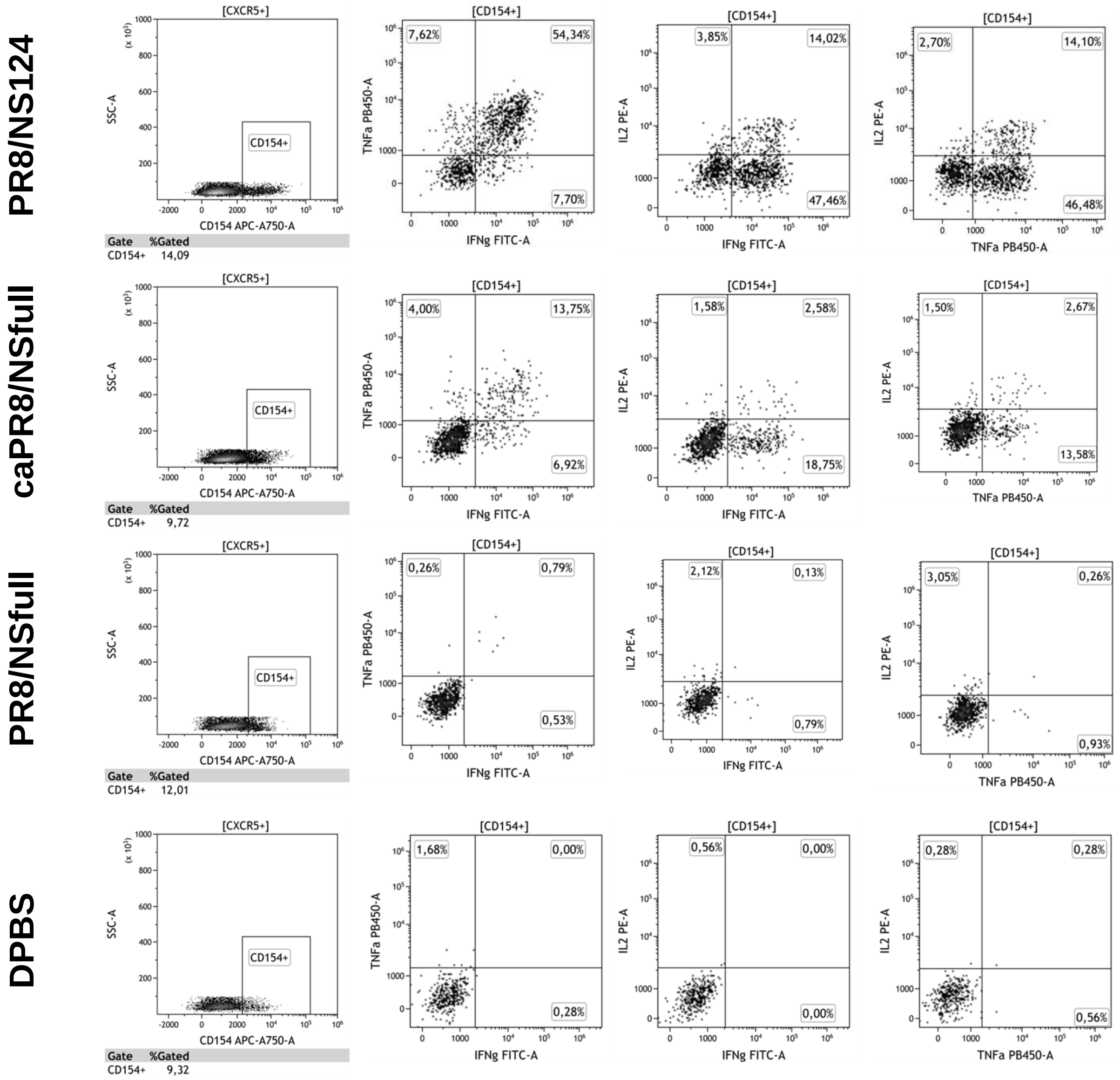

Figure S5. Representative plots of Tfh cells producing cytokines

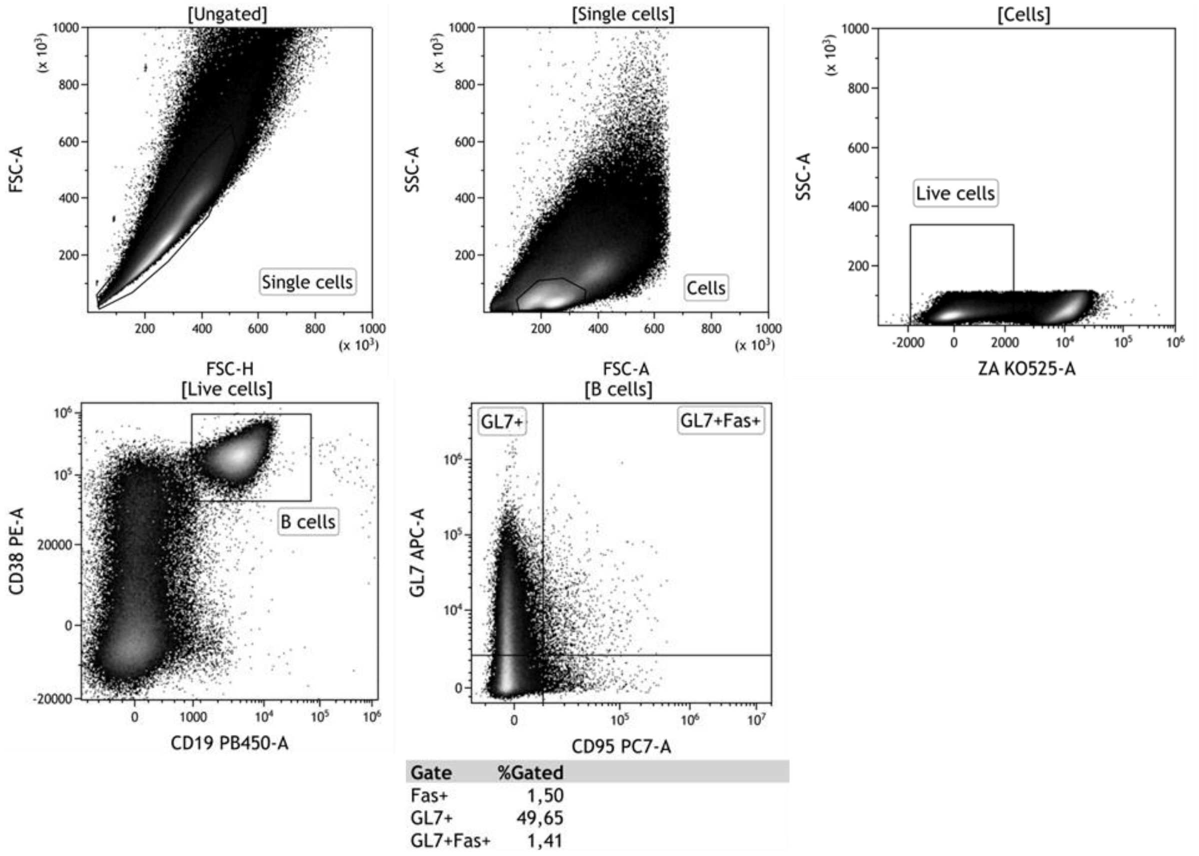

Figure S6. GC B-cells gaiting strategy. Non-viable cells are excluded from the analysis based on forward and side scattered light (FSC/SSC) parameters and the degree of binding of the viability marker Zombie Aqua. GC B-cells were identified as GL7+Fas+ cells within the CD19+CD38+ subpopulation.

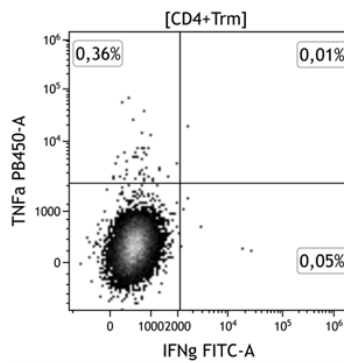

Supplement: Supplementary file 1 [file vaccines-13-00058-s001.zip › vaccines-3379565-supplementary.pdf]
